# Supplementary material for: Are the predicted known bacterial strains in a sample really present? A case study
Source: PLoS One. 2023 Oct 13;18(10):e0291964. doi: 10.1371/journal.pone.0291964 (PMC10575510; doi:10.1371/journal.pone.0291964)
Supplement: S1 Table — (DOCX) [file pone.0291964.s001.docx]

**Supplementary Table S1. Known strain unique SNP coverages.**

| Known strains | | | Uniform Distribution (p-value) | #original SNPs | #unique SNP | #unique SNPs within reads | %unique SNPs within reads | #unique SNPs without reads | %unique SNPs without reads | Average coverage |
| --- | --- | --- | --- | --- | --- | --- | --- | --- | --- | --- |
| *S. aureus* | Chng | CN1 | 9.7E-34 | 21016 | 3210 | 70 | 2.2 | 3140 | 97.8 | 36.4 |
|  |  | JH1 | 0.03 | 24308 | 882 | 13 | 1.5 | 869 | 98.5 | 13.8 |
|  |  | MSSA476 | 1.0E-17 | 20886 | 3321 | 40 | 1.2 | 3281 | 98.8 | 25.5 |
|  |  | ST398 | 4.9E-156 | 54316 | 35753 | 265 | 0.7 | 35488 | 99.3 | 41 |
|  | Pathoscope2 | CIGC93 | 4.7E-86 | 19194 | 3055 | 100 | 3.3 | 2955 | 96.7 | 17.2 |
|  |  | NN54 | 0.01 | 23597 | 889 | 4 | 0.5 | 885 | 99.6 | 15.6 |
|  |  | USA300_TCH959 | 7.1E-100 | 20802 | 3515 | 153 | 4.4 | 3362 | 95.6 | 19.1 |
|  | StrainEst | sa21196 | 6.0E-27 | 20890 | 3847 | 65 | 1.7 | 3782 | 98.3 | 23.3 |
|  |  | sa21343 | 1.6E-22 | 22939 | 4483 | 37 | 0.8 | 4446 | 99.2 | 32.4 |
|  |  | CIGC93 | 4.7E-86 | 19194 | 3055 | 100 | 3.3 | 2955 | 96.7 | 17.2 |
|  |  | USA300_TCH959 | 7.1E-100 | 20802 | 3515 | 153 | 4.4 | 3362 | 95.6 | 19.1 |
| *S. epidermidis* | Chng | NIHLM023 | 0 | 59716 | 9969 | 5839 | 58.6 | 4130 | 41.4 | 26.8 |
|  |  | NIHLM039 | 4.2E-208 | 9837 | 1557 | 453 | 29.1 | 1104 | 70.9 | 48.8 |
|  |  | SK135 | 2.5E-20 | 6918 | 424 | 31 | 7.3 | 393 | 92.7 | 81.2 |
|  |  | VCU129 | 0 | 61716 | 11406 | 8731 | 76.5 | 2675 | 23.5 | 30.4 |
|  | Pathoscope2 | SK135 | 2.5E-20 | 6918 | 424 | 31 | 7.3 | 393 | 92.7 | 81.2 |
|  |  | VCU129 | 0 | 61716 | 11406 | 8731 | 76.5 | 2675 | 23.5 | 30.4 |
|  | StrainEst | NIH051668 | 0 | 8661 | 2376 | 791 | 33.3 | 1585 | 66.7 | 24.7 |
|  |  | NIHLM023 | 0 | 59716 | 9969 | 5839 | 58.6 | 4130 | 41.4 | 26.8 |
|  |  | VCU109 | 1.3E-47 | 7662 | 458 | 108 | 23.6 | 350 | 76.4 | 25 |
|  |  | VCU123 | 2.7E-152 | 8845 | 762 | 227 | 29.8 | 535 | 70.2 | 25.8 |
|  |  | VCU129 | 0 | 61716 | 11406 | 8731 | 76.5 | 2675 | 23.5 | 30.4 |
