# Supplementary material for: Are the predicted known bacterial strains in a sample really present? A case study
Source: PLoS One. 2023 Oct 13;18(10):e0291964. doi: 10.1371/journal.pone.0291964 (PMC10575510; doi:10.1371/journal.pone.0291964)
Supplement: S2 Table — (DOCX) [file pone.0291964.s002.docx]

**Supplementary Table S2. Known strain correlations.**

| Known strains | | | #correlations | #significant correlations | %significant correlations |
| --- | --- | --- | --- | --- | --- |
| *S. aureus* | Chng | CN1 | 2415 | 391 | 16.2 |
|  |  | JH1 | 78 | 25 | 32.1 |
|  |  | MSSA476 | 780 | 114 | 14.6 |
|  |  | ST398 | 34980 | 3914 | 11.2 |
|  | Pathoscope2 | CIGC93 | 4950 | 1037 | 21.0 |
|  |  | NN54 | 6 | 4 | 66.7 |
|  |  | USA300_TCH959 | 11628 | 9408 | 80.9 |
|  | StrainEst | sa21196 | 2080 | 392 | 18.9 |
|  |  | sa21343 | 666 | 169 | 25.4 |
|  |  | CIGC93 | 4950 | 1037 | 21.0 |
|  |  | USA300_TCH959 | 11628 | 9408 | 80.9 |
| *S. epidermidis* | Chng | NIHLM023 | 499500 | 223381 | 44.7 |
|  |  | NIHLM039 | 102378 | 7653 | 7.5 |
|  |  | SK135 | 465 | 92 | 19.8 |
|  |  | VCU129 | 499500 | 56698 | 11.4 |
|  | Pathoscope2 | SK135 | 465 | 92 | 19.8 |
|  |  | VCU129 | 499500 | 56698 | 11.4 |
|  | StrainEst | NIH051668 | 312445 | 52493 | 16.8 |
|  |  | NIHLM023 | 499500 | 223381 | 44.7 |
|  |  | VCU109 | 5778 | 359 | 6.2 |
|  |  | VCU123 | 25651 | 3565 | 13.9 |
|  |  | VCU129 | 499500 | 56698 | 11.4 |
