# Supplementary material for: Are the predicted known bacterial strains in a sample really present? A case study
Source: PLoS One. 2023 Oct 13;18(10):e0291964. doi: 10.1371/journal.pone.0291964 (PMC10575510; doi:10.1371/journal.pone.0291964)
Supplement: S3 Table — (DOCX) [file pone.0291964.s003.docx]

**Supplementary Table S3. Strains used in database.**

| **S. aureus* (Total: 209) | taxid:904732\|Staphylococcus_aureus_subsp._aureus_21202 | <https://ftp.ncbi.nlm.nih.gov/genomes/all/GCF/000/239/655/GCF_000239655.1_ASM23965v2> |
| --- | --- | --- |
|  | taxid:904730\|Staphylococcus_aureus_subsp._aureus_21200 | <https://ftp.ncbi.nlm.nih.gov/genomes/all/GCF/000/221/825/GCF_000221825.1_ASM22182v2> |
|  | taxid:904727\|Staphylococcus_aureus_subsp._aureus_21194 | <https://ftp.ncbi.nlm.nih.gov/genomes/all/GCF/000/239/535/GCF_000239535.1_ASM23953v2> |
|  | taxid:1323661\|Staphylococcus_aureus_CA-347 | <https://ftp.ncbi.nlm.nih.gov/genomes/all/GCF/000/412/775/GCF_000412775.1_ASM41277v1> |
|  | taxid:931454\|Staphylococcus_aureus_subsp._aureus_CIG1524 | <https://ftp.ncbi.nlm.nih.gov/genomes/all/GCF/000/248/915/GCF_000248915.1_ASM24891v2> |
|  | taxid:553583\|Staphylococcus_aureus_A9635 | <https://ftp.ncbi.nlm.nih.gov/genomes/all/GCF/000/174/575/GCF_000174575.1_ASM17457v1> |
|  | taxid:931451\|Staphylococcus_aureus_subsp._aureus_CIG290 | <https://ftp.ncbi.nlm.nih.gov/genomes/all/GCF/000/248/855/GCF_000248855.1_ASM24885v2> |
|  | taxid:904743\|Staphylococcus_aureus_subsp._aureus_21252 | <https://ftp.ncbi.nlm.nih.gov/genomes/all/GCF/000/242/475/GCF_000242475.1_ASM24247v2> |
|  | taxid:585151\|Staphylococcus_aureus_subsp._aureus_C427 | <https://ftp.ncbi.nlm.nih.gov/genomes/all/GCF/000/162/615/GCF_000162615.1_ASM16261v1> |
|  | taxid:904772\|Staphylococcus_aureus_subsp._aureus_21342 | <https://ftp.ncbi.nlm.nih.gov/genomes/all/GCF/000/245/575/GCF_000245575.1_ASM24557v2> |
|  | taxid:585150\|Staphylococcus_aureus_subsp._aureus_C160 | <https://ftp.ncbi.nlm.nih.gov/genomes/all/GCF/000/162/855/GCF_000162855.1_ASM16285v1> |
|  | taxid:1163749\|Staphylococcus_aureus_subsp._aureus_LCT-SA112 | <https://ftp.ncbi.nlm.nih.gov/genomes/all/GCF/000/261/165/GCF_000261165.1_ASM26116v1> |
|  | taxid:904774\|Staphylococcus_aureus_subsp._aureus_21345 | <https://ftp.ncbi.nlm.nih.gov/genomes/all/GCF/000/245/615/GCF_000245615.1_ASM24561v2> |
|  | taxid:585161\|Staphylococcus_aureus_subsp._aureus_WW2703_97 | <https://ftp.ncbi.nlm.nih.gov/genomes/all/GCF/000/162/735/GCF_000162735.1_ASM16273v1> |
|  | taxid:585147\|Staphylococcus_aureus_subsp._aureus_A017934_97 | <https://ftp.ncbi.nlm.nih.gov/genomes/all/GCF/000/163/335/GCF_000163335.1_ASM16333v1> |
|  | taxid:904746\|Staphylococcus_aureus_subsp._aureus_21264 | <https://ftp.ncbi.nlm.nih.gov/genomes/all/GCF/000/242/515/GCF_000242515.1_ASM24251v2> |
|  | taxid:543538\|Staphylococcus_aureus_subsp._aureus_CGS00 | <https://ftp.ncbi.nlm.nih.gov/genomes/all/GCF/000/193/835/GCF_000193835.1_ASM19383v2> |
|  | taxid:548470\|Staphylococcus_aureus_subsp._aureus_MN8 | <https://ftp.ncbi.nlm.nih.gov/genomes/all/GCF/000/160/195/GCF_000160195.1_ASM16019v1> |
|  | taxid:585148\|Staphylococcus_aureus_subsp._aureus_Btn1260 | <https://ftp.ncbi.nlm.nih.gov/genomes/all/GCF/000/162/835/GCF_000162835.1_ASM16283v1> |
|  | taxid:282458\|Staphylococcus_aureus_subsp._aureus_MRSA252 | <https://ftp.ncbi.nlm.nih.gov/genomes/all/GCF/000/011/505/GCF_000011505.1_ASM1150v1> |
|  | taxid:585154\|Staphylococcus_aureus_subsp._aureus_EMRSA16 | <https://ftp.ncbi.nlm.nih.gov/genomes/all/GCF/000/163/575/GCF_000163575.1_ASM16357v1> |
|  | taxid:931436\|Staphylococcus_aureus_subsp._aureus_CIG1242 | <https://ftp.ncbi.nlm.nih.gov/genomes/all/GCF/000/248/555/GCF_000248555.2_ASM24855v3> |
|  | taxid:931453\|Staphylococcus_aureus_subsp._aureus_CIG149 | <https://ftp.ncbi.nlm.nih.gov/genomes/all/GCF/000/248/895/GCF_000248895.1_ASM24889v2> |
|  | taxid:931438\|Staphylococcus_aureus_subsp._aureus_CIG1605 | <https://ftp.ncbi.nlm.nih.gov/genomes/all/GCF/000/248/595/GCF_000248595.1_ASM24859v2> |
|  | taxid:931435\|Staphylococcus_aureus_subsp._aureus_CIG1214 | <https://ftp.ncbi.nlm.nih.gov/genomes/all/GCF/000/248/535/GCF_000248535.1_ASM24853v2> |
|  | taxid:931452\|Staphylococcus_aureus_subsp._aureus_CIG1267 | <https://ftp.ncbi.nlm.nih.gov/genomes/all/GCF/000/248/875/GCF_000248875.1_ASM24887v2> |
|  | taxid:931445\|Staphylococcus_aureus_subsp._aureus_CIG1176 | <https://ftp.ncbi.nlm.nih.gov/genomes/all/GCF/000/248/735/GCF_000248735.1_ASM24873v2> |
|  | taxid:931456\|Staphylococcus_aureus_subsp._aureus_CIGC341D | <https://ftp.ncbi.nlm.nih.gov/genomes/all/GCF/000/248/955/GCF_000248955.1_ASM24895v2> |
|  | taxid:931437\|Staphylococcus_aureus_subsp._aureus_CIG1500 | <https://ftp.ncbi.nlm.nih.gov/genomes/all/GCF/000/248/575/GCF_000248575.1_ASM24857v2> |
|  | taxid:931447\|Staphylococcus_aureus_subsp._aureus_CIG1233 | <https://ftp.ncbi.nlm.nih.gov/genomes/all/GCF/000/248/775/GCF_000248775.1_ASM24877v2> |
|  | taxid:904728\|Staphylococcus_aureus_subsp._aureus_21195 | <https://ftp.ncbi.nlm.nih.gov/genomes/all/GCF/000/221/845/GCF_000221845.1_ASM22184v2> |
|  | taxid:585160\|Staphylococcus_aureus_subsp._aureus_WBG10049 | <https://ftp.ncbi.nlm.nih.gov/genomes/all/GCF/000/162/715/GCF_000162715.1_ASM16271v1> |
|  | taxid:548473\|Staphylococcus_aureus_subsp._aureus_TCH60 | <https://ftp.ncbi.nlm.nih.gov/genomes/all/GCF/000/159/535/GCF_000159535.2_ASM15953v2> |
|  | taxid:585143\|Staphylococcus_aureus_subsp._aureus_55_2053 | <https://ftp.ncbi.nlm.nih.gov/genomes/all/GCF/000/160/335/GCF_000160335.2_ASM16033v2> |
|  | taxid:585153\|Staphylococcus_aureus_subsp._aureus_E1410 | <https://ftp.ncbi.nlm.nih.gov/genomes/all/GCF/000/160/395/GCF_000160395.1_ASM16039v1> |
|  | taxid:585146\|Staphylococcus_aureus_subsp._aureus_68-397 | <https://ftp.ncbi.nlm.nih.gov/genomes/all/GCF/000/160/375/GCF_000160375.1_ASM16037v1> |
|  | taxid:585144\|Staphylococcus_aureus_subsp._aureus_58-424 | <https://ftp.ncbi.nlm.nih.gov/genomes/all/GCF/000/162/815/GCF_000162815.1_ASM16281v1> |
|  | taxid:585145\|Staphylococcus_aureus_subsp._aureus_65-1322 | <https://ftp.ncbi.nlm.nih.gov/genomes/all/GCF/000/160/355/GCF_000160355.1_ASM16035v1> |
|  | taxid:585158\|Staphylococcus_aureus_subsp._aureus_M876 | <https://ftp.ncbi.nlm.nih.gov/genomes/all/GCF/000/160/415/GCF_000160415.1_ASM16041v1> |
|  | taxid:585159\|Staphylococcus_aureus_subsp._aureus_M899 | <https://ftp.ncbi.nlm.nih.gov/genomes/all/GCF/000/162/695/GCF_000162695.1_ASM16269v1> |
|  | taxid:585157\|Staphylococcus_aureus_subsp._aureus_M809 | <https://ftp.ncbi.nlm.nih.gov/genomes/all/GCF/000/162/795/GCF_000162795.1_ASM16279v1> |
|  | taxid:585156\|Staphylococcus_aureus_subsp._aureus_M1015 | <https://ftp.ncbi.nlm.nih.gov/genomes/all/GCF/000/162/675/GCF_000162675.1_ASM16267v1> |
|  | taxid:585149\|Staphylococcus_aureus_subsp._aureus_C101 | <https://ftp.ncbi.nlm.nih.gov/genomes/all/GCF/000/162/595/GCF_000162595.1_ASM16259v1> |
|  | taxid:904766\|Staphylococcus_aureus_subsp._aureus_21331 | <https://ftp.ncbi.nlm.nih.gov/genomes/all/GCF/000/239/555/GCF_000239555.1_ASM23955v2> |
|  | taxid:1155079\|Staphylococcus_aureus_subsp._aureus_DR10 | <https://ftp.ncbi.nlm.nih.gov/genomes/all/GCF/000/252/405/GCF_000252405.1_ASM25240v2> |
|  | taxid:1155084\|Staphylococcus_aureus_subsp._aureus_71193 | <https://ftp.ncbi.nlm.nih.gov/genomes/all/GCF/000/258/685/GCF_000258685.1_ASM25868v1> |
|  | taxid:523796\|Staphylococcus_aureus_subsp._aureus_ST398 | <https://ftp.ncbi.nlm.nih.gov/genomes/all/GCF/000/009/585/GCF_000009585.1_ASM958v1> |
|  | taxid:1229492\|Staphylococcus_aureus_08BA02176 | <https://ftp.ncbi.nlm.nih.gov/genomes/all/GCF/000/296/595/GCF_000296595.1_ASM29659v1> |
|  | taxid:585152\|Staphylococcus_aureus_subsp._aureus_D139 | <https://ftp.ncbi.nlm.nih.gov/genomes/all/GCF/000/162/635/GCF_000162635.1_ASM16263v1> |
|  | taxid:585155\|Staphylococcus_aureus_subsp._aureus_H19 | <https://ftp.ncbi.nlm.nih.gov/genomes/all/GCF/008/763/165/GCF_008763165.1_ASM876316v1> |
|  | taxid:869816\|Staphylococcus_aureus_subsp._aureus_JKD6159 | <https://ftp.ncbi.nlm.nih.gov/genomes/all/GCF/000/144/955/GCF_000144955.1_ASM14495v1> |
|  | taxid:948563\|Staphylococcus_aureus_O46 | <https://ftp.ncbi.nlm.nih.gov/genomes/all/GCF/000/189/455/GCF_000189455.2_ASM18945v3> |
|  | taxid:948561\|Staphylococcus_aureus_O11 | <https://ftp.ncbi.nlm.nih.gov/genomes/all/GCA/000/189/435/GCA_000189435.3_ASM18943v3> |
|  | taxid:904745\|Staphylococcus_aureus_subsp._aureus_21262 | <https://ftp.ncbi.nlm.nih.gov/genomes/all/GCF/000/242/495/GCF_000242495.1_ASM24249v2> |
|  | taxid:1392476\|Staphylococcus_aureus_subsp._aureus_6850 | <https://ftp.ncbi.nlm.nih.gov/genomes/all/GCF/000/462/955/GCF_000462955.1_ASM46295v1> |
|  | taxid:904786\|Staphylococcus_aureus_subsp._aureus_IS-160 | <https://ftp.ncbi.nlm.nih.gov/genomes/all/GCF/000/260/035/GCF_000260035.1_SaureusIS160v1.0> |
|  | taxid:1194085\|Staphylococcus_aureus_subsp._aureus_SA40 | <https://ftp.ncbi.nlm.nih.gov/genomes/all/GCF/000/470/865/GCF_000470865.1_ASM47086v1> |
|  | taxid:1147034\|Staphylococcus_aureus_PM1 | <https://ftp.ncbi.nlm.nih.gov/genomes/all/GCF/000/308/895/GCF_000308895.1_ASM30889v1> |
|  | taxid:1118959\|Staphylococcus_aureus_subsp._aureus_M013 | <https://ftp.ncbi.nlm.nih.gov/genomes/all/GCF/000/237/125/GCF_000237125.2_ASM23712v3> |
|  | taxid:1201010\|Staphylococcus_aureus_subsp._aureus_SA957 | <https://ftp.ncbi.nlm.nih.gov/genomes/all/GCF/000/470/845/GCF_000470845.1_ASM47084v1> |
|  | taxid:273036\|Staphylococcus_aureus_RF122 | <https://ftp.ncbi.nlm.nih.gov/genomes/all/GCF/000/009/005/GCF_000009005.1_ASM900v1> |
|  | taxid:985006\|Staphylococcus_aureus_subsp._aureus_LGA251 | <https://ftp.ncbi.nlm.nih.gov/genomes/all/GCF/000/237/265/GCF_000237265.1_ASM23726v1> |
|  | taxid:685039\|Staphylococcus_aureus_subsp._aureus_ED133 | <https://ftp.ncbi.nlm.nih.gov/genomes/all/GCF/000/210/315/GCF_000210315.1_ASM21031v1> |
|  | taxid:904738\|Staphylococcus_aureus_subsp._aureus_21235 | <https://ftp.ncbi.nlm.nih.gov/genomes/all/GCF/000/221/725/GCF_000221725.1_ASM22172v2> |
|  | taxid:904749\|Staphylococcus_aureus_subsp._aureus_21269 | <https://ftp.ncbi.nlm.nih.gov/genomes/all/GCF/000/221/745/GCF_000221745.1_ASM22174v2> |
|  | taxid:1074252\|Staphylococcus_aureus_subsp._aureus_HO_5096_0412 | <https://ftp.ncbi.nlm.nih.gov/genomes/all/GCF/000/284/535/GCF_000284535.1_ASM28453v1> |
|  | taxid:904760\|Staphylococcus_aureus_subsp._aureus_21310 | <https://ftp.ncbi.nlm.nih.gov/genomes/all/GCF/000/215/425/GCF_000215425.1_ASM21542v2> |
|  | taxid:904781\|Staphylococcus_aureus_subsp._aureus_IS-105 | <https://ftp.ncbi.nlm.nih.gov/genomes/all/GCF/000/247/375/GCF_000247375.1_ASM24737v2> |
|  | taxid:904773\|Staphylococcus_aureus_subsp._aureus_21343 | <https://ftp.ncbi.nlm.nih.gov/genomes/all/GCF/000/245/595/GCF_000245595.1_ASM24559v2> |
|  | taxid:450394\|Staphylococcus_aureus_subsp._aureus_USA300_TCH959 | <https://ftp.ncbi.nlm.nih.gov/genomes/all/GCF/000/153/665/GCF_000153665.1_ASM15366v1> |
|  | taxid:1280\|Staphylococcus_aureus_VH221/NCBI TaxID: 1278074 | <https://ftp.ncbi.nlm.nih.gov/genomes/all/GCF/000/364/485/GCF_000364485.1_S.aureus_VH221> |
|  | taxid:1144272\|Staphylococcus_aureus_subsp._aureus_GR1 | <https://ftp.ncbi.nlm.nih.gov/genomes/all/GCF/000/280/745/GCF_000280745.1_S.aureus_GR1_1.0> |
|  | taxid:548474\|Staphylococcus_aureus_subsp._aureus_TCH130 | <https://ftp.ncbi.nlm.nih.gov/genomes/all/GCF/000/159/555/GCF_000159555.1_ASM15955v1> |
|  | taxid:1193576\|Staphylococcus_aureus_subsp._aureus_CN1 | <https://ftp.ncbi.nlm.nih.gov/genomes/all/GCF/000/463/055/GCF_000463055.1_ASM46305v1> |
|  | taxid:904744\|Staphylococcus_aureus_subsp._aureus_21259 | <https://ftp.ncbi.nlm.nih.gov/genomes/all/GCA/000/221/785/GCA_000221785.2_ASM22178v2> |
|  | taxid:1182756\|Staphylococcus_aureus_subsp._aureus_str._Newbould_305 | <https://ftp.ncbi.nlm.nih.gov/genomes/all/GCF/000/276/625/GCF_000276625.1_Newbould305_assembly_v1> |
|  | taxid:904729\|Staphylococcus_aureus_subsp._aureus_21196 | <https://ftp.ncbi.nlm.nih.gov/genomes/all/GCF/000/332/685/GCF_000332685.1_Saur21196v1.0> |
|  | taxid:904777\|Staphylococcus_aureus_subsp._aureus_IS-55 | <https://ftp.ncbi.nlm.nih.gov/genomes/all/GCA/000/247/315/GCA_000247315.2_ASM24731v2> |
|  | taxid:904768\|Staphylococcus_aureus_subsp._aureus_21334 | <https://ftp.ncbi.nlm.nih.gov/genomes/all/GCF/000/239/575/GCF_000239575.1_ASM23957v2> |
|  | taxid:1221451\|Staphylococcus_aureus_subsp._aureus_120 | <https://ftp.ncbi.nlm.nih.gov/genomes/all/GCF/000/336/335/GCF_000336335.1_S.aureus_120> |
|  | taxid:1231382\|Staphylococcus_aureus_KT_Y21 | <https://ftp.ncbi.nlm.nih.gov/genomes/all/GCF/000/336/595/GCF_000336595.1_KTY21Sta1.0> |
|  | taxid:1221452\|Staphylococcus_aureus_subsp._aureus_333 | <https://ftp.ncbi.nlm.nih.gov/genomes/all/GCF/020/388/735/GCF_020388735.1_ASM2038873v1> |
|  | taxid:1226740\|Staphylococcus_aureus_subsp._aureus_VH60 | <https://ftp.ncbi.nlm.nih.gov/genomes/all/GCF/000/336/295/GCF_000336295.1_S.aureus_60> |
|  | taxid:1221453\|Staphylococcus_aureus_subsp._aureus_3989 | <https://ftp.ncbi.nlm.nih.gov/genomes/all/GCF/000/336/315/GCF_000336315.1_S.aureus_3989> |
|  | taxid:1144271\|Staphylococcus_aureus_subsp._aureus_118 | <https://ftp.ncbi.nlm.nih.gov/genomes/all/GCF/001/061/135/GCF_001061135.1_ASM106113v1> |
|  | taxid:1235277\|Staphylococcus_aureus_PPUKM-377-2009 | <https://ftp.ncbi.nlm.nih.gov/genomes/all/GCA/000/313/025/GCA_000313025.1_Staaur_19> |
|  | taxid:680649\|Staphylococcus_aureus_subsp._aureus_MR1 | <https://ftp.ncbi.nlm.nih.gov/genomes/all/GCF/000/176/195/GCF_000176195.1_ASM17619v1> |
|  | taxid:681288\|Staphylococcus_aureus_subsp._aureus_ED98 | <https://ftp.ncbi.nlm.nih.gov/genomes/all/GCF/000/024/585/GCF_000024585.1_ASM2458v1> |
|  | taxid:931444\|Staphylococcus_aureus_subsp._aureus_CIG1150 | <https://ftp.ncbi.nlm.nih.gov/genomes/all/GCF/000/248/715/GCF_000248715.1_ASM24871v2> |
|  | taxid:904750\|Staphylococcus_aureus_subsp._aureus_21272 | <https://ftp.ncbi.nlm.nih.gov/genomes/all/GCF/000/242/535/GCF_000242535.1_ASM24253v2> |
|  | taxid:904783\|Staphylococcus_aureus_subsp._aureus_IS-122 | <https://ftp.ncbi.nlm.nih.gov/genomes/all/GCF/000/247/415/GCF_000247415.1_ASM24741v2> |
|  | taxid:553581\|Staphylococcus_aureus_A9299 | <https://ftp.ncbi.nlm.nih.gov/genomes/all/GCF/000/174/555/GCF_000174555.1_ASM17455v1> |
|  | taxid:931455\|Staphylococcus_aureus_subsp._aureus_CIGC340D | <https://ftp.ncbi.nlm.nih.gov/genomes/all/GCF/000/248/935/GCF_000248935.1_ASM24893v2> |
|  | taxid:505321\|Staphylococcus_aureus_subsp._aureus_str._CF-Marseille | <https://ftp.ncbi.nlm.nih.gov/genomes/all/GCA/000/180/395/GCA_000180395.1_ASM18039v1> |
|  | taxid:1116216\|Staphylococcus_aureus_subsp._aureus_VRS3a | <https://ftp.ncbi.nlm.nih.gov/genomes/all/GCF/000/262/855/GCF_000262855.1_VRSA03_3> |
|  | taxid:1161392\|Staphylococcus_aureus_NN54 | <https://ftp.ncbi.nlm.nih.gov/genomes/all/GCF/000/308/915/GCF_000308915.1_ASM30891v1> |
|  | taxid:158879\|Staphylococcus_aureus_subsp._aureus_N315 | <https://ftp.ncbi.nlm.nih.gov/genomes/all/GCF/000/009/645/GCF_000009645.1_ASM964v1> |
|  | taxid:418127\|Staphylococcus_aureus_subsp._aureus_Mu3 | <https://ftp.ncbi.nlm.nih.gov/genomes/all/GCF/000/010/445/GCF_000010445.1_ASM1044v1> |
|  | taxid:585891\|Staphylococcus_aureus_subsp._aureus_Mu50-omega | <https://ftp.ncbi.nlm.nih.gov/genomes/all/GCF/000/180/095/GCF_000180095.1_ASM18009v1> |
|  | taxid:158878\|Staphylococcus_aureus_subsp._aureus_Mu50 | <https://ftp.ncbi.nlm.nih.gov/genomes/all/GCF/000/009/665/GCF_000009665.1_ASM966v1> |
|  | taxid:904731\|Staphylococcus_aureus_subsp._aureus_21201 | <https://ftp.ncbi.nlm.nih.gov/genomes/all/GCF/000/221/805/GCF_000221805.1_ASM22180v2> |
|  | taxid:904763\|Staphylococcus_aureus_subsp._aureus_21318 | <https://ftp.ncbi.nlm.nih.gov/genomes/all/GCA/000/215/445/GCA_000215445.2_ASM21544v2> |
|  | taxid:904739\|Staphylococcus_aureus_subsp._aureus_21236 | <https://ftp.ncbi.nlm.nih.gov/genomes/all/GCF/000/332/665/GCF_000332665.1_Saur21236v1.0> |
|  | taxid:553573\|Staphylococcus_aureus_A8115 | <https://ftp.ncbi.nlm.nih.gov/genomes/all/GCF/000/174/535/GCF_000174535.1_ASM17453v1> |
|  | taxid:553574\|Staphylococcus_aureus_A8117 | <https://ftp.ncbi.nlm.nih.gov/genomes/all/GCF/000/175/955/GCF_000175955.1_ASM17595v1> |
|  | taxid:1074919\|Staphylococcus_aureus_subsp._aureus_ST228 | <https://ftp.ncbi.nlm.nih.gov/genomes/all/GCF/900/038/245/GCF_900038245.1_7554_6_49> |
|  | taxid:1134842\|Staphylococcus_aureus_subsp._aureus_CM05 | <https://ftp.ncbi.nlm.nih.gov/genomes/all/GCF/000/294/385/GCF_000294385.2_ASM29438v2> |
|  | taxid:889933\|Staphylococcus_aureus_subsp._aureus_ECT-R_2 | <https://ftp.ncbi.nlm.nih.gov/genomes/all/GCF/000/253/135/GCF_000253135.1_ASM25313v1> |
|  | taxid:553565\|Staphylococcus_aureus_A5937 | <https://ftp.ncbi.nlm.nih.gov/genomes/all/GCF/000/174/455/GCF_000174455.1_ASM17445v1> |
|  | taxid:931432\|Staphylococcus_aureus_subsp._aureus_CIG1057 | <https://ftp.ncbi.nlm.nih.gov/genomes/all/GCF/000/248/475/GCF_000248475.1_ASM24847v2> |
|  | taxid:931434\|Staphylococcus_aureus_subsp._aureus_CIG1165 | <https://ftp.ncbi.nlm.nih.gov/genomes/all/GCF/000/248/515/GCF_000248515.1_ASM24851v2> |
|  | taxid:904723\|Staphylococcus_aureus_subsp._aureus_21172 | <https://ftp.ncbi.nlm.nih.gov/genomes/all/GCF/000/205/365/GCF_000205365.1_ASM20536v2> |
|  | taxid:543540\|Staphylococcus_aureus_subsp._aureus_CGS03 | <https://ftp.ncbi.nlm.nih.gov/genomes/all/GCF/000/193/875/GCF_000193875.1_ASM19387v2> |
|  | taxid:931458\|Staphylococcus_aureus_subsp._aureus_CIGC348 | <https://ftp.ncbi.nlm.nih.gov/genomes/all/GCF/000/248/995/GCF_000248995.1_ASM24899v2> |
|  | taxid:1116218\|Staphylococcus_aureus_subsp._aureus_VRS5 | <https://ftp.ncbi.nlm.nih.gov/genomes/all/GCF/000/262/895/GCF_000262895.1_VRSA05_2> |
|  | taxid:1116220\|Staphylococcus_aureus_subsp._aureus_VRS7 | <https://ftp.ncbi.nlm.nih.gov/genomes/all/GCF/000/262/935/GCF_000262935.1_VRSA07_3> |
|  | taxid:359787\|Staphylococcus_aureus_subsp._aureus_JH1 | Current update in NCBI as of 22 July 2022: record removed; assembly suppressed; <https://www.ncbi.nlm.nih.gov/assembly/GCF_000017125.1/>; at the time of analysis this was still available and theres was no indication of record being removed. |
|  | taxid:359786\|Staphylococcus_aureus_subsp._aureus_JH9 | <https://ftp.ncbi.nlm.nih.gov/genomes/all/GCF/000/016/805/GCF_000016805.1_ASM1680v1> |
|  | taxid:1158696\|Staphylococcus_aureus_M0770 | <https://ftp.ncbi.nlm.nih.gov/genomes/all/GCF/000/361/945/GCF_000361945.1_Stap_aure_M0770_V1> |
|  | taxid:553577\|Staphylococcus_aureus_A8796 | <https://ftp.ncbi.nlm.nih.gov/genomes/all/GCF/000/178/015/GCF_000178015.1_ASM17801v1> |
|  | taxid:1158694\|Staphylococcus_aureus_M0719 | <https://ftp.ncbi.nlm.nih.gov/genomes/all/GCF/000/361/905/GCF_000361905.1_Stap_aure_M0719_V1> |
|  | taxid:553580\|Staphylococcus_aureus_A8819 | <https://ftp.ncbi.nlm.nih.gov/genomes/all/GCF/000/178/035/GCF_000178035.1_ASM17803v1> |
|  | taxid:1116224\|Staphylococcus_aureus_subsp._aureus_VRS11a | <https://ftp.ncbi.nlm.nih.gov/genomes/all/GCF/000/263/015/GCF_000263015.1_VRSA11a_2> |
|  | taxid:1116225\|Staphylococcus_aureus_subsp._aureus_VRS11b | <https://ftp.ncbi.nlm.nih.gov/genomes/all/GCF/000/263/035/GCF_000263035.1_VRSA11b_2> |
|  | taxid:904775\|Staphylococcus_aureus_subsp._aureus_IS-3 | <https://ftp.ncbi.nlm.nih.gov/genomes/all/GCF/000/247/275/GCF_000247275.1_ASM24727v2> |
|  | taxid:1116217\|Staphylococcus_aureus_subsp._aureus_VRS4 | <https://ftp.ncbi.nlm.nih.gov/genomes/all/GCF/000/262/875/GCF_000262875.1_VRSA04_3> |
|  | taxid:553596\|Staphylococcus_aureus_A9781 | <https://ftp.ncbi.nlm.nih.gov/genomes/all/GCF/000/174/635/GCF_000174635.1_ASM17463v1> |
|  | taxid:553588\|Staphylococcus_aureus_A9719 | <https://ftp.ncbi.nlm.nih.gov/genomes/all/GCF/000/174/595/GCF_000174595.1_ASM17459v1> |
|  | taxid:1158695\|Staphylococcus_aureus_M0769 | <https://ftp.ncbi.nlm.nih.gov/genomes/all/GCF/000/361/925/GCF_000361925.1_Stap_aure_M0769_V1> |
|  | taxid:1158494\|Staphylococcus_aureus_M1451 | <https://ftp.ncbi.nlm.nih.gov/genomes/all/GCF/000/363/525/GCF_000363525.1_Stap_aure_M1451_V1> |
|  | taxid:904792\|Staphylococcus_aureus_subsp._aureus_IS-M | <https://ftp.ncbi.nlm.nih.gov/genomes/all/GCF/000/257/985/GCF_000257985.1_SaureusISMv1.0> |
|  | taxid:1116221\|Staphylococcus_aureus_subsp._aureus_VRS8 | <https://ftp.ncbi.nlm.nih.gov/genomes/all/GCF/000/262/955/GCF_000262955.1_VRSA08_2> |
|  | taxid:931446\|Staphylococcus_aureus_subsp._aureus_CIG1213 | <https://ftp.ncbi.nlm.nih.gov/genomes/all/GCF/000/248/755/GCF_000248755.1_ASM24875v2> |
|  | taxid:931443\|Staphylococcus_aureus_subsp._aureus_CIG1096 | <https://ftp.ncbi.nlm.nih.gov/genomes/all/GCF/000/248/695/GCF_000248695.1_ASM24869v2> |
|  | taxid:703339\|Staphylococcus_aureus_04-02981 | <https://ftp.ncbi.nlm.nih.gov/genomes/all/GCF/000/025/145/GCF_000025145.1_ASM2514v1> |
|  | taxid:553568\|Staphylococcus_aureus_A6224 | <https://ftp.ncbi.nlm.nih.gov/genomes/all/GCF/000/174/495/GCF_000174495.1_ASM17449v1> |
|  | taxid:553601\|Staphylococcus_aureus_A10102 | <https://ftp.ncbi.nlm.nih.gov/genomes/all/GCF/000/175/495/GCF_000175495.1_ASM17549v1> |
|  | taxid:553571\|Staphylococcus_aureus_A6300 | <https://ftp.ncbi.nlm.nih.gov/genomes/all/GCF/000/174/515/GCF_000174515.1_ASM17451v1> |
|  | taxid:931439\|Staphylococcus_aureus_subsp._aureus_CIG1750 | <https://ftp.ncbi.nlm.nih.gov/genomes/all/GCF/000/248/615/GCF_000248615.1_ASM24861v2> |
|  | taxid:931440\|Staphylococcus_aureus_subsp._aureus_CIG1769 | <https://ftp.ncbi.nlm.nih.gov/genomes/all/GCF/000/248/635/GCF_000248635.1_ASM24863v2> |
|  | taxid:553592\|Staphylococcus_aureus_A9763 | <https://ftp.ncbi.nlm.nih.gov/genomes/all/GCF/000/174/615/GCF_000174615.1_ASM17461v1> |
|  | taxid:1116219\|Staphylococcus_aureus_subsp._aureus_VRS6 | <https://ftp.ncbi.nlm.nih.gov/genomes/all/GCF/000/262/915/GCF_000262915.1_VRSA06_2> |
|  | taxid:1116214\|Staphylococcus_aureus_subsp._aureus_VRS1 | <https://ftp.ncbi.nlm.nih.gov/genomes/all/GCF/000/262/815/GCF_000262815.1_VRSA01_3> |
|  | taxid:1116215\|Staphylococcus_aureus_subsp._aureus_VRS2 | <https://ftp.ncbi.nlm.nih.gov/genomes/all/GCF/000/262/835/GCF_000262835.1_VRSA02_3> |
|  | taxid:1116222\|Staphylococcus_aureus_subsp._aureus_VRS9 | <https://ftp.ncbi.nlm.nih.gov/genomes/all/GCF/000/262/975/GCF_000262975.1_VRSA09_2> |
|  | taxid:1116223\|Staphylococcus_aureus_subsp._aureus_VRS10 | <https://ftp.ncbi.nlm.nih.gov/genomes/all/GCF/000/262/995/GCF_000262995.1_VRSA10_2> |
|  | taxid:904726\|Staphylococcus_aureus_subsp._aureus_21193 | <https://ftp.ncbi.nlm.nih.gov/genomes/all/GCF/000/205/385/GCF_000205385.1_ASM20538v2> |
|  | taxid:904759\|Staphylococcus_aureus_subsp._aureus_21305 | <https://ftp.ncbi.nlm.nih.gov/genomes/all/GCF/000/215/405/GCF_000215405.1_ASM21540v2> |
|  | taxid:904771\|Staphylococcus_aureus_subsp._aureus_21340 | <https://ftp.ncbi.nlm.nih.gov/genomes/all/GCF/000/239/595/GCF_000239595.1_ASM23959v2> |
|  | taxid:762962\|Staphylococcus_aureus_subsp._aureus_ATCC_51811 | <https://ftp.ncbi.nlm.nih.gov/genomes/all/GCF/000/164/715/GCF_000164715.1_ASM16471v1> |
|  | taxid:931459\|Staphylococcus_aureus_subsp._aureus_CIGC128 | <https://ftp.ncbi.nlm.nih.gov/genomes/all/GCF/000/249/015/GCF_000249015.1_ASM24901v2> |
|  | taxid:282459\|Staphylococcus_aureus_subsp._aureus_MSSA476 | <https://ftp.ncbi.nlm.nih.gov/genomes/all/GCF/000/011/525/GCF_000011525.1_ASM1152v1> |
|  | taxid:1231383\|Staphylococcus_aureus_KT_314250 | <https://ftp.ncbi.nlm.nih.gov/genomes/all/GCF/000/336/515/GCF_000336515.1_KT3Sta1.0> |
|  | taxid:196620\|Staphylococcus_aureus_subsp._aureus_MW2 | <https://ftp.ncbi.nlm.nih.gov/genomes/all/GCF/000/011/265/GCF_000011265.1_ASM1126v1> |
|  | taxid:548475\|Staphylococcus_aureus_subsp._aureus_TCH70 | <https://ftp.ncbi.nlm.nih.gov/genomes/all/GCF/000/149/015/GCF_000149015.1_ASM14901v1> |
|  | taxid:931441\|Staphylococcus_aureus_subsp._aureus_CIG1835 | <https://ftp.ncbi.nlm.nih.gov/genomes/all/GCF/000/248/655/GCF_000248655.1_ASM24865v2> |
|  | taxid:931460\|Staphylococcus_aureus_subsp._aureus_CIGC93 | <https://ftp.ncbi.nlm.nih.gov/genomes/all/GCF/000/249/035/GCF_000249035.1_ASM24903v2> |
|  | taxid:904313\|Staphylococcus_aureus_subsp._aureus_VCU006 | <https://ftp.ncbi.nlm.nih.gov/genomes/all/GCF/000/260/315/GCF_000260315.1_ASM26031v1> |
|  | taxid:546343\|Staphylococcus_aureus_subsp._aureus_str._JKD6009 | <https://ftp.ncbi.nlm.nih.gov/genomes/all/GCF/900/607/245/GCF_900607245.1_JKD6009> |
|  | taxid:546342\|Staphylococcus_aureus_subsp._aureus_str._JKD6008 | <https://ftp.ncbi.nlm.nih.gov/genomes/all/GCF/000/145/595/GCF_000145595.1_ASM14559v1> |
|  | taxid:1006543\|Staphylococcus_aureus_subsp._aureus_T0131 | <https://ftp.ncbi.nlm.nih.gov/genomes/all/GCF/000/204/665/GCF_000204665.1_ASM20466v1> |
|  | taxid:908247\|Staphylococcus_aureus_16K | <https://ftp.ncbi.nlm.nih.gov/genomes/all/GCF/000/208/695/GCF_000208695.1_ASM20869v1> |
|  | taxid:1321369\|Staphylococcus_aureus_Bmb9393 | <https://ftp.ncbi.nlm.nih.gov/genomes/all/GCF/000/418/345/GCF_000418345.1_ASM41834v1> |
|  | taxid:862516\|Staphylococcus_aureus_subsp._aureus_ATCC_BAA-39 | <https://ftp.ncbi.nlm.nih.gov/genomes/all/GCF/000/146/385/GCF_000146385.1_ASM14638v1> |
|  | taxid:904785\|Staphylococcus_aureus_subsp._aureus_IS-157 | <https://ftp.ncbi.nlm.nih.gov/genomes/all/GCF/000/260/015/GCF_000260015.1_SaureusIS157v1.0> |
|  | taxid:904724\|Staphylococcus_aureus_subsp._aureus_21178 | <https://ftp.ncbi.nlm.nih.gov/genomes/all/GCF/000/239/635/GCF_000239635.1_ASM23963v2> |
|  | taxid:663951\|Staphylococcus_aureus_subsp._aureus_TW20 | <https://ftp.ncbi.nlm.nih.gov/genomes/all/GCF/002/224/955/GCF_002224955.1_ASM222495v1> |
|  | taxid:1406863\|Staphylococcus_aureus_subsp._aureus_Z172 | <https://ftp.ncbi.nlm.nih.gov/genomes/all/GCF/000/485/885/GCF_000485885.1_ASM48588v1> |
|  | taxid:904787\|Staphylococcus_aureus_subsp._aureus_IS-189 | <https://ftp.ncbi.nlm.nih.gov/genomes/all/GCA/000/260/055/GCA_000260055.1_SaureusIS189v1.0> |
|  | taxid:1235276\|Staphylococcus_aureus_PPUKM-332-2009 | <https://ftp.ncbi.nlm.nih.gov/genomes/all/GCA/000/312/985/GCA_000312985.1_Staaur_18> |
|  | taxid:1235278\|Staphylococcus_aureus_PPUKM-775-2009 | <https://ftp.ncbi.nlm.nih.gov/genomes/all/GCA/000/313/005/GCA_000313005.1_Staaur_20> |
|  | taxid:1235275\|Staphylococcus_aureus_PPUKM-261-2009 | <https://ftp.ncbi.nlm.nih.gov/genomes/all/GCA/000/312/965/GCA_000312965.1_Staaur_17> |
|  | taxid:93062\|Staphylococcus_aureus_subsp._aureus_COL | <https://ftp.ncbi.nlm.nih.gov/genomes/all/GCF/000/012/045/GCF_000012045.1_ASM1204v1> |
|  | taxid:426430\|Staphylococcus_aureus_subsp._aureus_str._Newman | <https://ftp.ncbi.nlm.nih.gov/genomes/all/GCF/000/010/465/GCF_000010465.1_ASM1046v1> |
|  | taxid:904753\|Staphylococcus_aureus_subsp._aureus_21282 | <https://ftp.ncbi.nlm.nih.gov/genomes/all/GCF/000/330/685/GCF_000330685.1_Saureus21282v1.0> |
|  | taxid:904754\|Staphylococcus_aureus_subsp._aureus_21283 | <https://ftp.ncbi.nlm.nih.gov/genomes/all/GCF/000/242/555/GCF_000242555.1_ASM24255v2> |
|  | taxid:553567\|Staphylococcus_aureus_A5948 | <https://ftp.ncbi.nlm.nih.gov/genomes/all/GCF/000/174/475/GCF_000174475.1_ASM17447v1> |
|  | taxid:1305598\|Staphylococcus_aureus_M1 | <https://ftp.ncbi.nlm.nih.gov/genomes/all/GCF/000/367/745/GCF_000367745.1_ASM36774v1> |
|  | taxid:931442\|Staphylococcus_aureus_subsp._aureus_CIG547 | <https://ftp.ncbi.nlm.nih.gov/genomes/all/GCF/000/248/675/GCF_000248675.1_ASM24867v2> |
|  | taxid:452948\|Staphylococcus_aureus_930918-3 | <https://ftp.ncbi.nlm.nih.gov/genomes/all/GCF/000/171/435/GCF_000171435.1_ASM17143v1> |
|  | taxid:553594\|Staphylococcus_aureus_A9765 | <https://ftp.ncbi.nlm.nih.gov/genomes/all/GCF/000/175/475/GCF_000175475.1_ASM17547v1> |
|  | taxid:931450\|Staphylococcus_aureus_subsp._aureus_CIG2018 | <https://ftp.ncbi.nlm.nih.gov/genomes/all/GCF/000/248/835/GCF_000248835.1_ASM24883v2> |
|  | taxid:904735\|Staphylococcus_aureus_subsp._aureus_21209 | <https://ftp.ncbi.nlm.nih.gov/genomes/all/GCF/000/239/675/GCF_000239675.1_ASM23967v2> |
|  | taxid:931448\|Staphylococcus_aureus_subsp._aureus_CIG1612 | <https://ftp.ncbi.nlm.nih.gov/genomes/all/GCF/000/248/795/GCF_000248795.1_ASM24879v2> |
|  | taxid:931433\|Staphylococcus_aureus_subsp._aureus_CIG1114 | <https://ftp.ncbi.nlm.nih.gov/genomes/all/GCF/000/248/495/GCF_000248495.1_ASM24849v2> |
|  | taxid:931449\|Staphylococcus_aureus_subsp._aureus_CIG1770 | <https://ftp.ncbi.nlm.nih.gov/genomes/all/GCF/000/248/815/GCF_000248815.1_ASM24881v2> |
|  | taxid:904737\|Staphylococcus_aureus_subsp._aureus_21232 | <https://ftp.ncbi.nlm.nih.gov/genomes/all/GCF/000/239/615/GCF_000239615.1_ASM23961v2> |
|  | taxid:644279\|Staphylococcus_aureus_subsp._aureus_132 | <https://ftp.ncbi.nlm.nih.gov/genomes/all/GCF/000/174/955/GCF_000174955.1_ASM17495v1> |
|  | taxid:455227\|Staphylococcus_aureus_D30 | <https://ftp.ncbi.nlm.nih.gov/genomes/all/GCF/000/171/455/GCF_000171455.1_ASM17145v1> |
|  | taxid:754025\|Staphylococcus_aureus_subsp._aureus_MRSA131 | <https://ftp.ncbi.nlm.nih.gov/genomes/all/GCF/000/187/145/GCF_000187145.1_ASM18714v1> |
|  | taxid:754026\|Staphylococcus_aureus_subsp._aureus_MRSA177 | <https://ftp.ncbi.nlm.nih.gov/genomes/all/GCF/000/187/165/GCF_000187165.1_ASM18716v1> |
|  | taxid:543539\|Staphylococcus_aureus_subsp._aureus_CGS01 | <https://ftp.ncbi.nlm.nih.gov/genomes/all/GCF/000/193/855/GCF_000193855.1_ASM19385v2> |
|  | taxid:451516\|Staphylococcus_aureus_subsp._aureus_USA300_TCH1516 | <https://ftp.ncbi.nlm.nih.gov/genomes/all/GCF/000/017/085/GCF_000017085.1_ASM1708v1> |
|  | taxid:904782\|Staphylococcus_aureus_subsp._aureus_IS-111 | <https://ftp.ncbi.nlm.nih.gov/genomes/all/GCF/000/247/395/GCF_000247395.1_ASM24739v2> |
|  | taxid:904778\|Staphylococcus_aureus_subsp._aureus_IS-88 | <https://ftp.ncbi.nlm.nih.gov/genomes/all/GCF/000/247/355/GCF_000247355.1_ASM24735v2> |
|  | taxid:451515\|Staphylococcus_aureus_subsp._aureus_USA300_FPR3757 | <https://ftp.ncbi.nlm.nih.gov/genomes/all/GCF/000/013/465/GCF_000013465.1_ASM1346v1> |
|  | taxid:553590\|Staphylococcus_aureus_A9754 | <https://ftp.ncbi.nlm.nih.gov/genomes/all/GCF/000/177/995/GCF_000177995.1_ASM17799v1> |
|  | taxid:931457\|Staphylococcus_aureus_subsp._aureus_CIGC345D | <https://ftp.ncbi.nlm.nih.gov/genomes/all/GCF/000/248/975/GCF_000248975.1_ASM24897v2> |
|  | taxid:1112349\|Staphylococcus_aureus_subsp._aureus_NN50 | <https://ftp.ncbi.nlm.nih.gov/genomes/all/GCF/000/284/895/GCF_000284895.1_ASM28489v1> |
|  | taxid:1241616\|Staphylococcus_aureus_subsp._aureus_DSM_20231 | <https://ftp.ncbi.nlm.nih.gov/genomes/all/GCF/001/027/105/GCF_001027105.1_ASM102710v1> |
|  | taxid:561307\|Staphylococcus_aureus_subsp._aureus_RN4220 | <https://ftp.ncbi.nlm.nih.gov/genomes/all/GCF/000/212/435/GCF_000212435.1_ASM21243v2> |
|  | taxid:1028799\|Staphylococcus_aureus_subsp._aureus_VC40 | <https://ftp.ncbi.nlm.nih.gov/genomes/all/GCF/000/245/495/GCF_000245495.1_ASM24549v1> |
|  | taxid:93061\|Staphylococcus_aureus_subsp._aureus_NCTC_8325 | <https://ftp.ncbi.nlm.nih.gov/genomes/all/GCF/000/013/425/GCF_000013425.1_ASM1342v1> |
|  | taxid:904725\|Staphylococcus_aureus_subsp._aureus_21189 | <https://ftp.ncbi.nlm.nih.gov/genomes/all/GCF/000/205/345/GCF_000205345.1_ASM20534v2> |
|  | taxid:1283278\|Staphylococcus_aureus_KLT6 | <https://ftp.ncbi.nlm.nih.gov/genomes/all/GCF/000/350/425/GCF_000350425.2_KLT6> |
|  | taxid:904747\|Staphylococcus_aureus_subsp._aureus_21266 | <https://ftp.ncbi.nlm.nih.gov/genomes/all/GCA/000/221/765/GCA_000221765.2_ASM22176v2> |
|  | taxid:904767\|Staphylococcus_aureus_subsp._aureus_21333 | <https://ftp.ncbi.nlm.nih.gov/genomes/all/GCF/000/242/575/GCF_000242575.1_ASM24257v2> |
|  | taxid:1123523\|Staphylococcus_aureus_subsp._aureus_11819-97 | <https://ftp.ncbi.nlm.nih.gov/genomes/all/GCF/000/239/235/GCF_000239235.1_ASM23923v1> |
| *S. epidermidis* (Total: 61) | taxid:1000590\|Staphylococcus_epidermidis_14.1.R1.SE | <https://ftp.ncbi.nlm.nih.gov/genomes/all/GCA/000/239/515/GCA_000239515.2_ASM23951v2> |
|  | taxid:1155130\|Staphylococcus_epidermidis_NIH051475 | <https://ftp.ncbi.nlm.nih.gov/genomes/all/GCF/000/275/965/GCF_000275965.1_ASM27596v1> |
|  | taxid:1155131\|Staphylococcus_epidermidis_NIH051668 | <https://ftp.ncbi.nlm.nih.gov/genomes/all/GCF/000/275/985/GCF_000275985.2_ASM27598v2> |
|  | taxid:1155132\|Staphylococcus_epidermidis_NIH04003 | <https://ftp.ncbi.nlm.nih.gov/genomes/all/GCF/000/276/005/GCF_000276005.1_ASM27600v1> |
|  | taxid:1155133\|Staphylococcus_epidermidis_NIH05003 | <https://ftp.ncbi.nlm.nih.gov/genomes/all/GCF/000/276/025/GCF_000276025.1_ASM27602v1> |
|  | taxid:1155134\|Staphylococcus_epidermidis_NIH06004 | <https://ftp.ncbi.nlm.nih.gov/genomes/all/GCF/000/276/045/GCF_000276045.1_ASM27604v1> |
|  | taxid:1155135\|Staphylococcus_epidermidis_NIH08001 | <https://ftp.ncbi.nlm.nih.gov/genomes/all/GCF/000/276/065/GCF_000276065.1_ASM27606v1> |
|  | taxid:1220510\|Staphylococcus_epidermidis_AU12_03 | <https://ftp.ncbi.nlm.nih.gov/genomes/all/GCF/000/308/395/GCF_000308395.1_ASM30839v1> |
|  | taxid:1235440\|Staphylococcus_epidermidis_12142587 | <https://ftp.ncbi.nlm.nih.gov/genomes/all/GCF/000/304/575/GCF_000304575.1_ASM30457v1> |
|  | taxid:176279\|Staphylococcus_epidermidis_RP62A | <https://ftp.ncbi.nlm.nih.gov/genomes/all/GCF/000/011/925/GCF_000011925.1_ASM1192v1> |
|  | taxid:176280\|Staphylococcus_epidermidis_ATCC_12228 | <https://ftp.ncbi.nlm.nih.gov/genomes/all/GCF/000/007/645/GCF_000007645.1_ASM764v1> |
|  | taxid:525374\|Staphylococcus_epidermidis_BCM_HMP0060 | <https://ftp.ncbi.nlm.nih.gov/genomes/all/GCF/000/159/575/GCF_000159575.1_ASM15957v1> |
|  | taxid:525375\|Staphylococcus_epidermidis_M23864:W2(grey) | <https://ftp.ncbi.nlm.nih.gov/genomes/all/GCF/000/164/075/GCF_000164075.1_ASM16407v1> |
|  | taxid:525376\|Staphylococcus_epidermidis_W23144 | <https://ftp.ncbi.nlm.nih.gov/genomes/all/GCF/000/160/235/GCF_000160235.1_ASM16023v1> |
|  | taxid:596317\|Staphylococcus_epidermidis_SK135 | <https://ftp.ncbi.nlm.nih.gov/genomes/all/GCF/000/177/115/GCF_000177115.1_ASM17711v1> |
|  | taxid:764544\|Staphylococcus_epidermidis_FRI909 | <https://ftp.ncbi.nlm.nih.gov/genomes/all/GCF/000/186/205/GCF_000186205.1_ASM18620v2> |
|  | taxid:883130\|Staphylococcus_epidermidis_BVS058A4 | <https://ftp.ncbi.nlm.nih.gov/genomes/all/GCF/000/314/715/GCF_000314715.2_Stap_epid_BVS058A4_V1> |
|  | taxid:904317\|Staphylococcus_epidermidis_VCU028 | <https://ftp.ncbi.nlm.nih.gov/genomes/all/GCF/000/205/405/GCF_000205405.1_ASM20540v2> |
|  | taxid:904319\|Staphylococcus_epidermidis_VCU037 | <https://ftp.ncbi.nlm.nih.gov/genomes/all/GCF/000/221/705/GCF_000221705.1_ASM22170v2> |
|  | taxid:904320\|Staphylococcus_epidermidis_VCU041 | <https://ftp.ncbi.nlm.nih.gov/genomes/all/GCF/000/245/635/GCF_000245635.1_ASM24563v2> |
|  | taxid:904321\|Staphylococcus_epidermidis_VCU045 | <https://ftp.ncbi.nlm.nih.gov/genomes/all/GCF/000/205/425/GCF_000205425.1_ASM20542v2> |
|  | taxid:904323\|Staphylococcus_epidermidis_VCU057 | <https://ftp.ncbi.nlm.nih.gov/genomes/all/GCA/000/245/655/GCA_000245655.2_ASM24565v2> |
|  | taxid:904324\|Staphylococcus_epidermidis_VCU065 | <https://ftp.ncbi.nlm.nih.gov/genomes/all/GCF/000/245/675/GCF_000245675.1_ASM24567v2> |
|  | taxid:904325\|Staphylococcus_epidermidis_VCU071 | <https://ftp.ncbi.nlm.nih.gov/genomes/all/GCF/000/260/295/GCF_000260295.1_ASM26029v1> |
|  | taxid:904326\|Staphylococcus_epidermidis_VCU081 | <https://ftp.ncbi.nlm.nih.gov/genomes/all/GCF/000/245/695/GCF_000245695.1_ASM24569v2> |
|  | taxid:904328\|Staphylococcus_epidermidis_VCU105 | <https://ftp.ncbi.nlm.nih.gov/genomes/all/GCF/000/221/685/GCF_000221685.1_ASM22168v2> |
|  | taxid:904330\|Staphylococcus_epidermidis_VCU109 | <https://ftp.ncbi.nlm.nih.gov/genomes/all/GCF/000/221/665/GCF_000221665.1_ASM22166v2> |
|  | taxid:904335\|Staphylococcus_epidermidis_VCU117 | <https://ftp.ncbi.nlm.nih.gov/genomes/all/GCF/000/247/025/GCF_000247025.1_ASM24702v2> |
|  | taxid:904336\|Staphylococcus_epidermidis_VCU118 | <https://ftp.ncbi.nlm.nih.gov/genomes/all/GCF/000/247/045/GCF_000247045.1_ASM24704v2> |
|  | taxid:904337\|Staphylococcus_epidermidis_VCU120 | <https://ftp.ncbi.nlm.nih.gov/genomes/all/GCF/000/247/065/GCF_000247065.1_ASM24706v2> |
|  | taxid:904340\|Staphylococcus_epidermidis_VCU123 | <https://ftp.ncbi.nlm.nih.gov/genomes/all/GCA/000/247/105/GCA_000247105.2_ASM24710v2> |
|  | taxid:904341\|Staphylococcus_epidermidis_VCU125 | <https://ftp.ncbi.nlm.nih.gov/genomes/all/GCF/000/247/125/GCF_000247125.1_ASM24712v2> |
|  | taxid:904342\|Staphylococcus_epidermidis_VCU126 | <https://ftp.ncbi.nlm.nih.gov/genomes/all/GCF/000/247/145/GCF_000247145.1_ASM24714v2> |
|  | taxid:904343\|Staphylococcus_epidermidis_VCU127 | <https://ftp.ncbi.nlm.nih.gov/genomes/all/GCF/000/247/165/GCF_000247165.1_ASM24716v2> |
|  | taxid:904344\|Staphylococcus_epidermidis_VCU128 | <https://ftp.ncbi.nlm.nih.gov/genomes/all/GCF/000/247/185/GCF_000247185.1_ASM24718v2> |
|  | taxid:904345\|Staphylococcus_epidermidis_VCU129 | <https://ftp.ncbi.nlm.nih.gov/genomes/all/GCF/000/247/205/GCF_000247205.1_ASM24720v2> |
|  | taxid:904347\|Staphylococcus_epidermidis_VCU144 | <https://ftp.ncbi.nlm.nih.gov/genomes/all/GCF/000/205/325/GCF_000205325.1_ASM20532v2> |
|  | taxid:979199\|Staphylococcus_epidermidis_NIHLM095 | <https://ftp.ncbi.nlm.nih.gov/genomes/all/GCF/000/276/545/GCF_000276545.1_ASM27654v1> |
|  | taxid:979200\|Staphylococcus_epidermidis_NIHLM088 | <https://ftp.ncbi.nlm.nih.gov/genomes/all/GCA/000/276/525/GCA_000276525.1_ASM27652v1> |
|  | taxid:979201\|Staphylococcus_epidermidis_NIHLM087 | <https://ftp.ncbi.nlm.nih.gov/genomes/all/GCF/000/276/505/GCF_000276505.1_ASM27650v1> |
|  | taxid:979202\|Staphylococcus_epidermidis_NIHLM070 | <https://ftp.ncbi.nlm.nih.gov/genomes/all/GCA/000/276/485/GCA_000276485.1_ASM27648v1> |
|  | taxid:979203\|Staphylococcus_epidermidis_NIHLM067 | <https://ftp.ncbi.nlm.nih.gov/genomes/all/GCF/000/276/465/GCF_000276465.1_ASM27646v1> |
|  | taxid:979204\|Staphylococcus_epidermidis_NIHLM061 | <https://ftp.ncbi.nlm.nih.gov/genomes/all/GCF/000/276/445/GCF_000276445.1_ASM27644v1> |
|  | taxid:979205\|Staphylococcus_epidermidis_NIHLM057 | <https://ftp.ncbi.nlm.nih.gov/genomes/all/GCF/000/276/425/GCF_000276425.1_ASM27642v1> |
|  | taxid:979206\|Staphylococcus_epidermidis_NIHLM053 | <https://ftp.ncbi.nlm.nih.gov/genomes/all/GCF/000/276/405/GCF_000276405.1_ASM27640v1> |
|  | taxid:979207\|Staphylococcus_epidermidis_NIHLM049 | <https://ftp.ncbi.nlm.nih.gov/genomes/all/GCF/000/276/385/GCF_000276385.1_ASM27638v1> |
|  | taxid:979208\|Staphylococcus_epidermidis_NIHLM040 | <https://ftp.ncbi.nlm.nih.gov/genomes/all/GCF/000/276/365/GCF_000276365.1_ASM27636v1> |
|  | taxid:979209\|Staphylococcus_epidermidis_NIHLM039 | <https://ftp.ncbi.nlm.nih.gov/genomes/all/GCF/000/276/345/GCF_000276345.1_ASM27634v1> |
|  | taxid:979210\|Staphylococcus_epidermidis_NIHLM037 | <https://ftp.ncbi.nlm.nih.gov/genomes/all/GCF/000/276/325/GCF_000276325.1_ASM27632v1> |
|  | taxid:979211\|Staphylococcus_epidermidis_NIHLM023 | <https://ftp.ncbi.nlm.nih.gov/genomes/all/GCF/000/276/305/GCF_000276305.1_ASM27630v1> |
|  | taxid:979212\|Staphylococcus_epidermidis_NIHLM021 | <https://ftp.ncbi.nlm.nih.gov/genomes/all/GCF/000/276/285/GCF_000276285.1_ASM27628v1> |
|  | taxid:979213\|Staphylococcus_epidermidis_NIHLM020 | <https://ftp.ncbi.nlm.nih.gov/genomes/all/GCF/000/276/265/GCF_000276265.1_ASM27626v1> |
|  | taxid:979214\|Staphylococcus_epidermidis_NIHLM031 | <https://ftp.ncbi.nlm.nih.gov/genomes/all/GCF/000/276/245/GCF_000276245.1_ASM27624v1> |
|  | taxid:979215\|Staphylococcus_epidermidis_NIHLM018 | <https://ftp.ncbi.nlm.nih.gov/genomes/all/GCF/000/276/225/GCF_000276225.1_ASM27622v1> |
|  | taxid:979216\|Staphylococcus_epidermidis_NIHLM015 | <https://ftp.ncbi.nlm.nih.gov/genomes/all/GCF/000/276/205/GCF_000276205.1_ASM27620v1> |
|  | taxid:979217\|Staphylococcus_epidermidis_NIHLM008 | <https://ftp.ncbi.nlm.nih.gov/genomes/all/GCF/000/276/185/GCF_000276185.1_ASM27618v1> |
|  | taxid:979218\|Staphylococcus_epidermidis_NIHLM003 | <https://ftp.ncbi.nlm.nih.gov/genomes/all/GCF/000/276/165/GCF_000276165.1_ASM27616v1> |
|  | taxid:979219\|Staphylococcus_epidermidis_NIHLM001 | <https://ftp.ncbi.nlm.nih.gov/genomes/all/GCF/000/276/145/GCF_000276145.1_ASM27614v1> |
|  | taxid:979220\|Staphylococcus_epidermidis_NIH05005 | <https://ftp.ncbi.nlm.nih.gov/genomes/all/GCF/000/276/125/GCF_000276125.1_ASM27612v1> |
|  | taxid:979221\|Staphylococcus_epidermidis_NIH05001 | <https://ftp.ncbi.nlm.nih.gov/genomes/all/GCF/000/276/105/GCF_000276105.1_ASM27610v1> |
|  | taxid:979222\|Staphylococcus_epidermidis_NIH04008 | <https://ftp.ncbi.nlm.nih.gov/genomes/all/GCF/000/276/085/GCF_000276085.1_ASM27608v1> |

******S. aureus* strains: IS-125, IS-24, IS-91 and CO-23 were not found in the assembly_summary.txt and were not included, while strain M2 was also not found during the time of analysis and so was also not included.

*S. aureus* (<https://ftp.ncbi.nlm.nih.gov/genomes/refseq/bacteria/Staphylococcus_aureus/assembly_summary.txt>) and *S. epidermidis* (<https://ftp.ncbi.nlm.nih.gov/genomes/refseq/bacteria/Staphylococcus_epidermidis/assembly_summary.txt>) strains used were usually screened from the corresponding assembly_summary.txt files for each species.
