# Supplementary material for: Are the predicted known bacterial strains in a sample really present? A case study
Source: PLoS One. 2023 Oct 13;18(10):e0291964. doi: 10.1371/journal.pone.0291964 (PMC10575510; doi:10.1371/journal.pone.0291964)
Supplement: Attachment — Submitted filename: Major revision required.pdf [file pone.0291964.s005.pdf]

The article entitled “Are known bacterial strains really present?” seems to be interesting for the research scientific community. In the revised current form authors have shown the good potential in improving this article, but still, it is in lack of structured. This reviewer thinks this article can be improve more by summarizing and pointing few concerns. Start from the abstracts, the provided abstracts could be further improved by incorporating the following elements: (1) The abstract should start with a clear and concise objective statement that highlights the specific research question or problem being addressed. This will help readers understand the purpose of the study right from the beginning. (2) Provide a brief description of the methods or approaches used in the study in one or two statements. This should include information on the data collection process, analysis techniques, and any specific tools used. (3) Instead of just stating that known strains cannot be predicted and are different across tools, provide more specific and quantitative information about the results obtained. This will provide the readers with a clearer understanding of the findings. (4) Discuss the broader implications of the study findings in a single statement. (5) Conclude the abstract by highlighting potential future directions or recommendations based on the study's outcomes. (6) In introduction, there is an excessive use of citations/references. In example, only first paragraph of the introduction including 14 references in 7 lines. Please use the citation/references according to the journal guidelines and where appropriate. (7) In introduction where stated “We reran Pathscope2 on these samples …” please clearly mention the validity of findings. Adding potential reasons for the discrepancies and significance of findings. This reviewer suggests to revise thoroughly the introduction section by mentioning the Objective statement with limitations and significances at the end of introduction; (8) Furthermore, why it is so important to use the data from study of Chng et. al, published in 2016. Please consider using some stats and data comparison from the recent scientific study from last 5 years. (9) This reviewer suggests to revise your references very carefully and according to the journal style. Most of the references used in this study are out dated. Please defend your research innovation by comparing the study from the reference list of last five years research. (10) Please revise the manuscript carefully, and avoid the grammar mistakes. I suggest you to invite a native speaker to proofread this article before further submission, or use any official English language proofread services.

Best of Luck!
