# Supplementary material for: Are the predicted known bacterial strains in a sample really present? A case study
Source: PLoS One. 2023 Oct 13;18(10):e0291964. doi: 10.1371/journal.pone.0291964 (PMC10575510; doi:10.1371/journal.pone.0291964)
Supplement: Attachment — Submitted filename: KnownStrainsResponse.docx [file pone.0291964.s004.docx]

Title: Are known bacterial strains really present?

Authors: Minerva Ventolero, Saidi Wang, Haiyan Hu, Xiaoman Li

Dear Dr. Achraf El Allali,

Thank you so much for the opportunity to revise the manuscript! We appreciate the constructive comments and suggestions from the reviewers. We have improved our manuscript to clarify the points which were not described clearly or properly, and responded to the points raised by the reviewers below and addressed them in the manuscript. We hope the modified manuscript can be accepted.

Best Regards,

Xiaoman Li

**Additional Editor Comments:**

In addition to the reviewer’s concerns, I would ask that you give further information about current de novo bacterial strain identification directly from shotgun metagenomic reads and what approaches can be taken to do this. The discussion section is very short and will benefit from a discussion of how current de novo approaches can help alleviate the problem addressed in the paper. Please make sure you answer all the concerns for further consideration of the paper.

**Response:** Thank you for the suggestion. We have edited the manuscript to include a brief discussion on de novo approaches for strain identification.

**Reviewer 1**

**Comment 1:** The manuscript “Are known bacterial strains really present?” is good but this reviewer has some major concerns. We know the facts that the bacterial genomes are higly mutated; In bacterial genomes there are certain regions which are variable and some are constant so variability arisis and henece different strains; The analysis depends upon the use of databases used for either alignment or annotation for identification. Probably the authors should try to use different databases; why these comments?- as I am not having data analysis files. so unable to comment. If you have them please check for - how much variability is there from the original analysis; What is being used (Databases) initially for analysis. Keep the fact that the data, databases and information is growing and available day by day, so some variability is expected but within limits; The tools Pathoscope and IsoRead are used for the analysis, so right use of databases is very important for any type of analysis; compare the discussion with (https://doi.org/10.3389/fcell.2021.736994) (DOI: 10.1111/jam.15469) (<https://doi.org/10.1007/s11356-022-22197-4>)

**Response:** Thanks for the great comments. Yes, you are right that the analysis of known strains depends on the quality of the known strain genomes (the database) used. The annotated genome sequences stored at NCBI are often considered as gold standard (for instance, Chng et al. used the genomes from NCBI as well), the quality of which is more reliable than the genome sequences stored elsewhere. This is why we choose the annotated genome sequences at NCBI instead of those at elsewhere for the known strain analysis. Moreover, because we wanted to recreate the analysis of the previous study by Chng et al. 2016, which also used the annotated strain genomes at NCBI, we tried our best to replicate the set-up as much as possible, making sure that all specifically identified strains from the analysis they performed and reported that were still available in NCBI GenBank were part of the list of strains included in the recreated list of the genomes.

About the variability issue, these genomes we used are the reference genomes stored at NCBI, which normally do not change across time. If you are talking about the difference between two strain genomes of the two species, there are about 0.8% to 2% positions are different between a S. aureus strain with the species reference genome, and 0.3% to 2.6% positions are different between a S. epidermidis strain with the species reference genome. In terms of the difference between the list of strain genomes we used and those Chng et al. used, our list of the strain genomes for the two species targeted included every reported strains by Chng et al. and more genomes than Chng et al.’s list. The strain genomes we used were all from the latest curated list of strain genomes by NCBI RefSeq.

Thanks for the several interesting papers you mentioned. We are not sure how the diversity issue discussed in these paper relate to what we considered here. We focused on two species and their strains in the shotgun metagenomic reads, and did not focus on the general composition of the metagenomic samples in this study. Again, we completely agree that the genome sequences used affect the analysis results. We thus used the last version of the more reliable NCBI refseq genomes. Genomes from other resources can be explored, which may be problematic here as we are not so sure about their qualities.

**Reviewer 2:**

This is an interesting study and the authors have suggested an interesting idea. The author emphasizes the drawbacks of utilizing known strains for studying bacterial genomes in shotgun metagenomic samples, as bacterial genomes are continually evolving, making it uncertain whether previously identified known strains can provide meaningful insights for understanding current samples. The paper is generally well structured. However, in my opinion the paper has some shortcomings in regard to the analyses :

1- Can you provide an explanation for why the preprocessing parameters in the original paper differ from those used by the authors?

**Response:** Thank you for the comment. The parameters are the same except the input strain genomes are not completely the same as those in the Chng et al’s study, because the old genome file used by the Pathoscope2 tool in Chng et al.’s study is not supported by NCBI anymore. In brief, we tried to recreate as best we could the analysis originally performed by Chng et al., 2016. Due to some challenges that were encountered when using the tool Pathoscope 2 that we have explained in detail in the methods Section, a slight modification was performed. The main problem was the file that Pathoscope 2 was programmed to use from NCBI and which was discontinued and no longer made available by NCBI, and so we used an alternative approach to create the database list but making sure that the genomes we used were still form NCBI like the Chng et al.’s study. We also made sure that all available genomes of strains reported by Chng et al. were included in the database we created and used for the analysis. The tool StrainEst was not used in the original study but was included in our study as another tool of similar approach for strain analysis identification for comparative purposes only in our study.

2- It's not clear if the data used was screened for human contamination, which could affect the results. If it was, please include this information in the methods section.

**Response:** Thank you for the insightful comment. For the data screening for human contamination, it is often for the purpose of understanding the general composition of a metagenomic sample. In this study, our goal is to see which known strain of the two species is present. We no longer performed the screening of human information as the tools are designed to map sequences to a specific set of strain genomes that are the target of the analysis. A human DNA fragment will in general not be mapped to a strain genome here. In fact, we blasted each of these strain genomes we used against the human hg19 genome and found one and only one segment of 30 base pairs long with the e-value smaller than 1.0 was found in one of the S. epidermis strain genomes. More importantly, to claim the presence of a strain depends on the occurrence of many of the DNA fragments of this strain in the reads, which will not be affected by these contaminant (otherwise, a strain genome of these two species have a large overlap with the human genome). We have provided the information accordingly.

3- I recommend revising the methods section, specifically the section on strain analysis.

**Response:** Thank you for the suggestion. We have edited the methods section on strain analysis according to suggested changes.
