# Supplementary material for: Are the predicted known bacterial strains in a sample really present? A case study
Source: PLoS One. 2023 Oct 13;18(10):e0291964. doi: 10.1371/journal.pone.0291964 (PMC10575510; doi:10.1371/journal.pone.0291964)
Supplement: Attachment — Submitted filename: Response to Reviewers.docx [file pone.0291964.s006.docx]

Title: Are known bacterial strains really present?

Authors: Minerva Ventolero, Saidi Wang, Haiyan Hu, Xiaoman Li

Dear Dr. Achraf El Allali,

Thank you so much for the opportunity to revise the manuscript! We appreciate the constructive comments and suggestions from the reviewers. We have improved our manuscript to clarify the points which were not described clearly or properly, and responded to the points raised by the reviewers below and addressed them in the manuscript. We hope the modified manuscript can be accepted.

Best Regards,

Xiaoman Li

**Reviewer 1**

**Comment 1:** The article entitled “Are known bacterial strains really present?” seems to be interesting for the research scientific community. In the revised current form authors have shown the good potential in improving this article, but still, it is in lack of structured. This reviewer thinks this article can be improve more by summarizing and pointing few concerns. Start from the abstracts, the provided abstracts could be further improved by incorporating the following elements: **(1)** The abstract should start with a clear and concise objective statement that highlights the specific research question or problem being addressed. This will help readers understand the purpose of the study right from the beginning. **(2)** Provide a brief description of the methods or approaches used in the study in one or two statements. This should include information on the data collection process, analysis techniques, and any specific tools used. **(3)** Instead of just stating that known strains cannot be predicted and are different across tools, provide more specific and quantitative information about the results obtained. This will provide the readers with a clearer understanding of the findings. **(4)** Discuss the broader implications of the study findings in a single statement. **(5)** Conclude the abstract by highlighting potential future directions or recommendations based on the study's outcomes.

**Response:** Thank you for the great comments! We modified the abstract as you suggested.

**Comment 2:** **(6)** In introduction, there is an excessive use of citations/references. In example, only first paragraph of the introduction including 14 references in 7 lines. Please use the citation/references according to the journal guidelines and where appropriate. **(7)** In introduction where stated “We reran Pathscope2 on these samples …” please clearly mention the validity of findings. Adding potential reasons for the discrepancies and significance of findings. This reviewer suggests to revise thoroughly the introduction section by mentioning the Objective statement with limitations and significances at the end of introduction;

**Response:** Thanks for the comments! We rephrased the first paragraph and removed the first right references as you suggested. Note that the first eight references represent different applications and advancement by the next generation sequencing, including ChIP-seq for histone, ChIP-seq for transcription factors, the interaction of transcription factors from ChIP-seq, Hi-C, RNA-seq, metagenomics, strain studies, etc. They are necessary for laymen to understand the broad field of NGS while not all closely related to bacterial strains. We believe that other references are necessary and closely related to bacterial strain studies. We also added some more recent references.

We reran Pathscope2 because the original study applied Pathscope2 to predict known strains on these samples, and we wanted to see whether the rerun of Pathscope2 would result in a similar set of known strains. Moreover, the pathscope2 and StrainEST tools are more widely used and kept updating after their publication. There are a couple of more recent tools, which are not well tested and cited. Moreover, some of them are not for general bacterial species or only study strains in certain local regions. For these reasons, we chose pathscope2 and StrainEST. The rationale to choose the Chng et al. samples and the two tools are also included in the Methods section. We also added a paragraph to make our objective in the last paragraph of the introduction easier to be understood. The objectives were to show that the available methods have a flawed logic in their design and cannot really prove the existence of known strains. We also pointed out the limitation and significance of our study.

**Comment 3:** **(8)** Furthermore, why it is so important to use the data from study of Chng et. al, published in 2016. Please consider using some stats and data comparison from the recent scientific study from last 5 years.

**Response:** Thanks for the great comments! In our opinion, the choice of the data does not matter much, as the next generation sequencing technology and their application are already quite mature by 2012, including many analysis tools. We chose the Chng data because we have an interest in *S. aureus* and *S. epidermis*. Moreover, there are a large number of their known strains available. We believe our conclusions should hold with other datasets, other bacterial species, and other tools used, because as implied in the paper, all available tools claim the presence of a known strain if a portion or all of its SNPs (k-mers, MLTSs, etc.) occur in reads, which may be wrong and misleading. For instance, the occurrence of all its SNPs in reads could be due to the presence of several unknown strains, whose SNPs include all SNPs of this known strain.

**Comment 4:** **(9)** This reviewer suggests to revise your references very carefully and according to the journal style. Most of the references used in this study are out dated. Please defend your research innovation by comparing the study from the reference list of last five years research.

**Response:** Thanks for the very good comments! While it is true that some of the references included in this paper are not within the last five years, they are no less relevant. For instance, the pathscope2 and strainEST are still the most widely cited tools for known strain identification of general bacterial species on the genome scale (i.e., not locally, not for a given set of species). Note that, although some references are published more than five years ago, our study is still novel, as it shows novel insights about the limitations of the current known strain identification tools that scientists do not realize. In brief, available tools claim the occurrence of a known strain based on the occurrence of its SNPs or others in reads, which does not guarantee the occurrence of this known strain at all. We probably need to directly apply the novel strain prediction tools and experimentally validate the existing of these strains by long read sequencing.

**Comment 5:** **(10)** Please revise the manuscript carefully, and avoid the grammar mistakes. I suggest you to invite a native speaker to proofread this article before further submission, or use any official English language proofread services.

**Response: Point (10):** Thank you for the suggestion. We carefully modified the manuscript and have two native speakers proofread the manuscript.

**Reviewer 2:**

**Comment 1:** The case study described aimed to answer the title question by revisiting the known strains of S. aureus and S. epidermidis in atopic dermatitis shotgun metagenomic samples. The findings of the study revealed that even using the same analysis tools, the previously reported known strains could not be predicted consistently in the same samples. Additionally, different tools gave different results. The idea of the study is interesting, but the authors do not address the title question. The title is quite general and needs a more detailed analysis before the article is accepted. The author uses only two bacterial species, S. aureus and S. epidermis, so the results cannot be generalized to all bacterial species. I suggest that the author perform a more comprehensive analysis that includes more species or change the title of the article.

**Response:** Thank you for the great comments! We modified the title to emphasize that this is a case study and its conclusion may need to further comprehensively validate with more species and datasets. As we commented above, the conclusions made with this datasets and two species should be valid for other species and datasets as well, especially for those fast evolving bacterial species.

**Comment 2:** Another concern is the scripts used: "<https://github.com/UCF-Li-Lab/Min_AD_project_code>" are not documented, so replication of the study is not possible.

**Response:** Thanks for the helpful comment. We updated the readme file and the link to help readers to replicate the analysis in this study.
